# An examination of fitness costs of glyphosate resistance in the common morning glory, *Ipomoea purpurea*

**DOI:** 10.1002/ece3.1776

**Published:** 2015-10-26

**Authors:** Catherine L. Debban, Sara Okum, Kathleen E. Pieper, Ariana Wilson, Regina S. Baucom

**Affiliations:** ^1^ Department of Biology University of Virginia 229 Gilmer Hall Charlottesville Virginia 22904; ^2^ Biological Sciences Department University of Cincinnati Cincinnati OH 45221; ^3^ Davison Life Sciences Building University of Georgia 120 East Green Street Athens Georgia 30602‐7223; ^4^ Department of Ecology and Evolutionary Biology University of Michigan 2059 Kraus Natural Science Building Ann Arbor Michigan 48103

**Keywords:** Defense, fitness costs, trade‐offs, weed

## Abstract

Fitness costs are frequently invoked to explain the presence of genetic variation underlying plant defense across many types of damaging agents. Despite the expectation that costs of resistance are prevalent, however, they have been difficult to detect in nature. To examine the potential that resistance confers a fitness cost, we examined the survival and fitness of genetic lines of the common morning glory, *Ipomoea purpurea*, that diverged in the level of resistance to the herbicide glyphosate. We planted a large field experiment and assessed survival following herbicide application as well as fitness of the divergent selection lines in the absence of the herbicide. We found that genetic lines selected for increased resistance exhibited lower death compared to control and susceptible lines in the presence of the herbicide, but no evidence that resistant lines produced fewer seeds in the absence of herbicide. However, susceptible lines produced more viable seeds than resistant or control lines, providing some evidence of a fitness cost in terms of seed germination, and thus potential empirical support for the expectation of trait trade‐offs as a consequence of adaptation to novel environments.

## Introduction

Fitness costs play a fundamental role in evolutionary and ecological theory and are frequently invoked to explain the presence of genetic variation underlying plant defense (Coley et al. 1985; Bergelson et al. 1996; Mauricio 1998). If there were no fitness costs of defense to herbivory, for example, then all individuals in a population should be maximally defended and variation in the population depleted (Rausher and Simms 1989; Mauricio 1998). Fitness costs of defense are thought to arise from resource limitations – in the absence of the damaging agent, a highly defended plant that allocates resources away from fitness and toward defense will be at a reproductive disadvantage relative to susceptible plants (Bazzaz et al. 1987; Nunez‐Farfan et al. 2007). The idea that life‐history trade‐offs constrain defense evolution has been investigated in a broad range of plant study systems and with different types of damaging agents – from biotic sources of damage such as herbivores and pathogens to abiotic sources such as frost and herbicide (Simms 1992; Simms and Triplett 1994; Mauricio 1998, 2000; Agrawal et al. 2004; Baucom and Mauricio 2004, 2008). Although the explanation for genetic variation that costs provide is intuitively appealing, costs associated with resistance are not universal, with one survey reporting fitness trade‐offs in only 25–50% of studies that examine resistance to herbicides, pathogens, and herbivory (Bergelson and Purrington 1996).

Researchers cite environmental and methodological reasons to explain the unexpectedly low frequency of costs – for example, Bergelson and Purrington found that greater control of the genetic background increases the probability of their detection (1996). A related but relatively unexamined possibility is the idea that trade‐offs of resistance are masked by variation in genes that have independent positive effects, such as genes that influence resource acquisition (van Noordwijk and de Jong 1986). If variation in genes that contribute to photosynthetic capability and nutrient acquisition positively influences both plant fitness and resistance, a trade‐off might not be identified in the absence of the selective agent, even if costs are present (van Noordwijk and de Jong 1986; Houle 1991). Another possibility is that selection has lowered the cost to an undetectable level by modifying the pleiotropic effects of resistance – such compensatory evolution has been documented in organisms exhibiting resistance to antibiotics and insecticide (Lenski 1998; ffrench‐Constant 2013) but has yet to be conclusively identified in an herbicide‐resistant species.

It has also been hypothesized that certain mechanisms of resistance are more likely to endow costs than others (Bergelson and Purrington 1996), and in support of this idea, a moderately greater number of herbicide resistance studies report costs (62%) relative to studies of resistance to pathogens (56%) and herbivores (29%, Bergelson and Purrington 1996). However, an alternative hypothesis – that this pattern could be driven by the mechanism of resistance to one particular herbicide class, the triazines (Bergelson and Purrington 1996) – appears to be very likely, with recent studies of newer classes of herbicide often reporting no evidence of fitness costs (*reviewed in* Vila‐Aiub et al. 2009; Délye et al. 2013a). This is striking for two reasons – one, costs are implicit to applied control efforts that utilize crop and herbicide rotations, and two, herbicides, like other xenobiotics, are incredibly strong selective agents used by basic scientists to study rapid adaptation. If costs are not detected from well‐designed experiments that can specifically control both the strength of selection and ubiquity of exposure *via* the selective agent, then perhaps the general expectation of costs associated with resistance should be treated more critically, and other explanations for the presence of genetic variation in natural systems need to be examined – for example*,* that variation underlying resistance may be transient and at nonequilibrium level such that directional selection will eventually move the population to a completely defended state (Rausher and Simms 1989).

Although the ability to control the selective agent in herbicide resistance studies would lend them to being ideal study systems for studying costs, many such examinations are performed with nonoptimal designs. Only 25% of studies that investigate costs of herbicide resistance control for genetic background effects (Vila‐Aiub et al. 2009) in spite of the suggestion, made almost 20 years ago, that experimentalists control for such effects (Bergelson and Purrington 1996). In addition, many herbicide resistance studies do not consider the number of generations that may have experienced exposure by the herbicide in the field, and thus when comparing field‐collected R to S types, are not controlling for the potential that compensatory evolution has occurred.

Two powerful experimental approaches for examining fitness costs are transgenic modification and artificial selection. The transformation of *Arabidopsis* with a mutant gene encoding acetolactate synthase conferred resistance to the herbicide chlorsulfuron and definitively identified a pleiotropic fitness cost of the allele – one that ultimately led to a 34% decline in fitness in the absence of the herbicide (Bergelson et al. 1996; ). A transgenic design is perhaps the best experimental strategy to identify costs; however, such strategy is experimentally restricted to model plant species and has yet to be replicated in an agricultural weed. Artificial selection, on the other hand, is a tool that has been broadly used in evolutionary ecology – from studies that examine the evolution of plant mating systems to studies that consider parasitoid and pesticide resistance in *Drosophila* and *Daphnia magna,* respectively (Worley and Barrett 2000; Kraaijeveld et al. 2002; Delph et al. 2004; Conner et al. 2011; Goldman and Travisano 2011; Jansen et al. 2011). Surprisingly, few herbicide resistance studies have used designs similar to those used in evolutionary ecology wherein randomly cross‐pollinated groups are utilized as controls and both increased and decreased resistance lines are generated. Replication of the selection program is likewise a concern – designs that use two or more replicate selection lines per direction of selection with multiple families in each replicate line, initially drawn from the same source population, would have the benefit of controlling the number of genetic backgrounds that contributed to divergent phenotypes and as well allow a distinction to be made between responses to selection and genetic drift.

Here, we use progeny generated from an artificial selection design to determine whether there are fitness costs associated with herbicide resistance in the common morning glory, *Ipomoea purpurea*. This species, which is both an ecological genetics model and a noxious agricultural weed (Baucom et al. 2011), exhibits resistance to the field‐rate application of glyphosate – the active ingredient in RoundUp, which is the most widely used herbicide in current‐day agriculture (Kuester et al. 2015). The evolutionary trajectory of resistance has previously been investigated in *I. purpurea* using a quantitative genetics framework, with the following general conclusions: genetic variation for this trait is present within this species (Baucom and Mauricio 2008), and, resistance, scored as a visual injury rating 2 weeks postglyphosate application, is under positive selection in field conditions (Baucom and Mauricio 2008). While these results show that the criteria for the evolution of a higher level of glyphosate resistance are met in this species (Baucom and Mauricio 2008), it is not known whether resistance carries a cost. Here, we address this gap in our knowledge by performing a field experiment to specifically ask the following: Do artificially evolved resistance lines exhibit a fitness benefit of resistance in the presence of herbicide in field conditions, and is there evidence for a cost of resistance in the absence of the herbicide? The results provided herein show that genetic lines selected for higher resistance in the greenhouse survive glyphosate application in the field compared to lines selected for decreased resistance, and that some, but not all components of fitness, are reduced in resistant families in the nonherbicide control environment.

## Methods

### Experimental system

*Ipomoea purpurea* (L.) Roth (Convolvulaceae), the common or tall morning glory, is often found in corn, cotton, and soybean fields in the southeast and midwestern USA as well as roadsides and waste areas. Seeds of this species germinate mid‐May through late August; flowering occurs 4–6 weeks postgermination and continues until the plants are killed by the first major frost. Individual plants bear multiple showy flowers per day (range: 0 to >80), and flowers are open for a single morning before senescing. Plants are prolific and are capable of producing as many as 8000 seeds per season (Chaney and Baucom 2014).

Glyphosate [*N*‐(phosphonomethyl)glycine] is the active ingredient in the nonspecific postemergence herbicide RoundUp. Glyphosate enters the plant by diffusion and moves into the plant phloem through either active or passive mechanisms (Shaner 2009). The herbicide translocates to the apical and root meristems where it functions by competitively inhibiting EPSP synthase, a key enzyme in the shikimate pathway (Franz et al. 1997). This pathway is responsible for synthesizing aromatic amino acids and secondary metabolites vital to plant growth and development (Tzin and Galili 2010); some estimates suggest that the shikimate pathway is responsible for 30% of a plant's total carbon (Maeda and Dudareva 2012).

### Germplasm collection and generation of selection lines

In the fall of 2000, seeds were randomly collected from a total of 122 individuals from the same population located at the University of Georgia's Plant Sciences Farm (Oconee Co., GA) and screened to identify the most and least resistant families as described by Baucom and Mauricio (i.e., those that exhibit the least/most stem die back following herbicide application; 2008). From this base population, the top and bottom 20% of families that were the most and least resistant (24 individuals each, low and high) were chosen to establish the following six lines for G1, the first generation of artificial selection: two increased resistance (hereafter R), two decreased resistance (hereafter S) and two control (C) lines. Each replicate selection line was comprised of 12 parents; individuals used in the control lines were chosen from the base population at random. Because control families were chosen at random from the entire base population, approximately 16% of families were also used as parents in either the R or S selection lines. This sampling ensured that the selection lines were begun from the same initial pool. Within the separate selection lines, each individual was crossed to an unrelated individual and was used as both the pollen and ovule parent, at least twice, for each “X” in Figure S1. This crossing design is similar to that of Worley and Barrett (2000) and has the advantage of creating a moderate number of families (16) in each selection line for the next generation. Flowers were emasculated the night before pollinations to prevent self‐pollination. On the day of pollination, anthers were taken from the pollen parent and touched to the stigma of the ovule parent and tagged accordingly.

We performed a progeny resistance assay with the seeds generated from this crossing design to identify parents for the next generation of selection (G2). Six replicates of each of the 96 families generated via crossing (or each “X” in Figure S1) were scarified and planted in a randomized design in the University of Cincinnati greenhouse, for a total of 576 experimental individuals. The plants were allowed to grow until approximately the 2–3 leaf stage, at which point measurements of growth (the height of the plant (cm) and the number of leaves) were taken. Measurements were recorded a second time when individuals were at the 4–5 leaf stage. After the second phenotyping, half of the experimental individuals or three replicates per family from each of the R/S/C selection lines were sprayed with 1.121 kg ai ha^−1^ of glyphosate using a CO_2_‐pressurized handheld sprayer (R and D Sprayers, Opelousas, LA). Approximately 2 weeks later, the following data were recorded: death, height (cm), the number of leaves present, and the number of leaves exhibiting damage. Our assay of resistance, and the character under direct artificial selection, is the proportion height of the vine remaining after herbicide application, or the height of the plant 2 weeks after herbicide spray standardized by its height immediately before spray. If plants maintained or continued to grow following treatment with the herbicide, they exhibited a “1” or >1 proportion height remaining after spray. If they were <1, they died back, and were affected by the herbicide application. Individuals that died as a result of the herbicide were given a “0” score for the proportion height remaining.

A second generation of artificial selection (G2) was performed using replicate full‐sibling seeds of the top and bottom 20% most and least resistant individuals identified from the R and S lines in the above G1 progeny resistance assay. We elected to use this “family selection” design (Falconer and MacKay 1996) as the most susceptible individuals often died in response to the herbicide, and we wanted to ensure that crosses of the R and S lines occurred at the same time and in the same greenhouse conditions. We again randomly chose individuals from families within the control lines to produce control progeny. All aspects of the crossing design were identical to that of the first generation of selection, and another progeny resistance assay, again identical to that presented above (*N* = 576 plants), was performed using seeds generated from the second round of selection. We generated seed for the field experiment using one additional round of artificial selection to produce G3 progeny. All aspects of the crossing design and mating were identical to the initial and second generations of selection described above. We avoided full‐ or half‐sibling matings in all generations of artificial selection, if possible. When not possible, we kept the number of such matings low and similar across selection lines so that the level of inbreeding would be approximately equal across lines. Inbreeding coefficients, estimated using the R package *pedigreemm* (Vazquez et al. 2010), did not differ across the selection lines (*F* = 0.265, *P* = 0.767), and the inbreeding coefficients (range, 0–0.156) did not exceed the level found in natural populations (i.e., 30%, Chang and Rausher 1998).

### Field experiment

To determine whether there were fitness benefits and/or costs of artificially evolved herbicide resistance in the field, we planted replicate progeny from the third generation of selection in a fenced and tilled agricultural field at the University of Cincinnati's Center for Field Studies in Harrison, OH, on 8 June 2012. We scarified four replicate seeds from each of 10 randomly chosen families from the six R/S/C selection lines, and planted them in a randomized design in two block/treatment combinations, for a total of 960 seeds planted among four experimental plots. One treatment served as the “no herbicide” environment, whereas plants in the other treatment were sprayed with herbicide. Experimental seeds were planted 0.75 m apart in a grid, and plants, once germinated, were provided a 1.2‐m stake to vine up and to help maintain identification of experimental individuals. Plants were allowed to grow for approximately 30 days – at which time we sprayed the herbicide treatment plots with 0.84 kg ai ha^−1^ of glyphosate using a CO_2_‐pressurized handheld sprayer (R and D Sprayers, Opelousas, LA). We used a slightly lower rate in the field than in the artificial selection in the greenhouse as our main interest was in the potential differences between selection lines in the presence and absence of herbicide, and preliminary work in the greenhouse indicated this rate would differentiate the artificial selection lines (a typical dose‐response experiment on this species utilizing herbicide rates ranging from 0 to 3.4 kg ai ha^−1^ is presented in Kuester et al. 2015). Prior to herbicide application, the following measurements were taken: height of the main stem of each plant, the number of leaves, and the length of each leaf (cm). All plots were weeded once thoroughly at the beginning of the experiment, but otherwise, natural vegetation was allowed to grow and compete with experimental individuals. Two weeks postglyphosate application, the following data were collected: death, the height of the main stem, number of leaves remaining, the number of damaged leaves, and the number of undamaged leaves. While we have previously examined variation in the proportion of the plant that is damaged as our operational estimate of resistance (Baucom and Mauricio 2008), here we focus on survival following herbicide application, and survival to produce flower and seed to be consistent with recent greenhouse assays (Kuester et al. 2015).

Plants in the no herbicide treatment began flowering 27 July 2012 and we recorded both the day of first flowering for each plant as well as the number of flowers three times a week until 29 August 2012 to determine whether there were differences among the selection lines in phenology and in early flower production. Plants in the herbicide treatment began flowering on 20 August 2012 and we recorded the day of first flowering for each plant as well as the number of flowers produced daily for a period of 2 months. We collected mature seeds from all plants during three complete rounds of collection, and following the first frost on 30 October 2012 we collected individual plants, put them in brown paper bags and brought them to the laboratory for processing where we harvested all seeds from each plant. We counted the number of seeds of each plant using the 750‐2 Total Count System seed counter (International Marketing and Design Co., San Antonio, TX). The precision and accuracy of the seed counter was verified previously (Chaney and Baucom 2014). Prior to analysis, individuals from either treatment environment that did not germinate (*N* = 102) or survive to herbicide application (*N* = 2) were removed from further analyses. One individual from the herbicide‐present environment was removed from the analysis due to a sprayer malfunction and our subsequent concern that the plant did not receive an herbicide dose that was consistent with the rest of the experimental individuals.

### Germination experiment

We next determined whether the artificial selection lines exhibited differences in the production of germinable seeds to determine whether lines selected for increased resistance exhibited a fitness cost through lower germination. We did so by performing two replicate germination assays in the laboratory using seeds collected from individuals from a single nonherbicide control plot (*N* = 240). In each germination assay, we randomly sampled 10 seeds from each maternal individual and plated them in Petri dishes filled with 10 mL of dH_2_O. Petri dishes were completely randomized on laboratory benches, and the number of seeds that germinated within 7 days was recorded as were the number of dead seeds (those that imbibed water and split open to reveal a dead embryo). We likewise recorded the number of seeds per Petri dish that were not obviously dead (i.e., did not imbibe water or germinate). Because the seeds assessed in the germination trials were open‐pollinated in the field, any difference in germination could be due to differences across maternal lines nested within selection replicate (*N* = 10), selection replicate nested within direction of selection (R_1_, R_2_, S_1_, S_2_, C_1_, C_2_) and/or the direction of selection (R/S/C).

### Data analysis

#### Benefits and costs of herbicide resistance in the field

To determine whether there is a benefit of artificially evolved herbicide resistance in field conditions, we examined survival 2 weeks postherbicide application and survival to produce flowers and seed. We were specifically interested in testing the hypotheses that more individuals from the increased resistance selection lines survived glyphosate application and survived to set seed. Furthermore, a benefit of herbicide resistance would also be apparent if individuals from resistant lines produced more seed than control and susceptible individuals in the presence of herbicide, and as such we examined total seed output of plants in the presence of herbicide. However, we caution that data on seed number from the herbicide‐present environment were highly non‐normal and thus did not hold to the assumptions of ANOVA.

#### Benefits, or fitness in the presence of herbicide

Using the *glmer* function of the *lme4* package (Bates et al. 2015) of the R statistical programming language (version 3.2.2; R Core Team 2015), we performed separate generalized linear mixed‐model logistic regressions to test the effects of direction of selection (R/S/C), replicate selection line (Line, nested within direction of selection) (R_1_, R_2_, S_1_, S_2_, C_1_, C_2_), full‐sibling family (hereafter “family”) nested within selection line (*N* = 10 per selection line), and block (*N* = 2), on survival postspray and survival to set seed in the herbicide‐present environment. We modeled the outcome of survival as a binary‐dependent variable (survival = 1) for survival postspray and ability to set flower and seed using the “binomial” option. We performed a mixed‐model analysis of variance using the *lmer* function of the *lme4* package (Bates et al. 2015) to examine total seed production in the presence of herbicide. This trait was log(*y* + 1) transformed prior to analysis to improve normality of the residuals.

#### Costs, or fitness in the absence of herbicide

To determine whether costs of herbicide resistance were present in lines artificially selected for increased resistance, we compared measures of fitness in the absence of herbicide – day of flowering, the number of flowers produced early in the season, the total number of seeds produced, and average seed weight – according to direction of selection (R/S/C). We were specifically interested in determining whether, in the absence of herbicide, individuals selected for increased resistance exhibited reduced fitness (i.e., fewer seeds) or differences in fitness correlates compared to control and susceptible individuals. For these analyses, we performed a mixed‐model analysis of variance using the *lmer* function of the *lme4* package (Bates et al. 2015). The total number of flowers, day of flowering, and average seed weight were log(*y* + 1) transformed prior to analysis to improve normality of the residuals. As in the assessment of benefits, we performed separate mixed‐model analysis of variance to test the effects of direction of selection (R/S/C), replicate selection line (nested within direction of selection) (R_1_, R_2_, etc.), family nested within selection line (*N* = 10 per selection line), and block (*N* = 2) on the total number of flowers and seeds produced in the absence of herbicide. In both the analysis of benefits and costs, family and replicate selection line were considered random effects in the model whereas block and direction of selection were considered fixed.

Finally, we assessed the potential that individuals from the increased resistance lines exhibited a cost in the form of lower germination by assessing both the proportion of germinable and dead seeds, respectively, in separate generalized linear models using the *glmer* function of the *lme4* package (Bates 2015). In these analyses, we used the proportion dead seeds or proportion seeds germinated as the dependent variable with direction of selection (R/S/C) and experimental replicate (*N* = 2) as fixed effects in the model. The selection line, nested within direction of selection, and family nested within selection line were considered random effects. We modeled both estimates of seed viability using the “binomial” option as above.

The significance of effects in all models were determined using a likelihood ratio test to compare the full model and the reduced model with the effect of interest removed; *P*‐values were determined with a chi‐squared test with one degree of freedom. To determine whether glyphosate response or fitness traits differed between the increased/decreased resistance lines and/or varied from the control lines (i.e., R vs. S; R vs. C, S vs. C), nonorthogonal pairwise comparisons were performed using the Welch's *t*‐test followed by *P*‐value adjustments for multiple comparisons using the Holm method in the p.adjust package in R (R Core Team 2015). These comparisons were likewise made with the dichotomous response variables in the herbicide‐present environment (survival, flower, and seed produced) and the dichotomous response variables in the herbicide‐absent environment (dead and germinable seeds) using G‐tests Zar 2007, again followed by *P*‐value adjustments.

## Results

### The benefit of herbicide resistance

**Figure 1:**
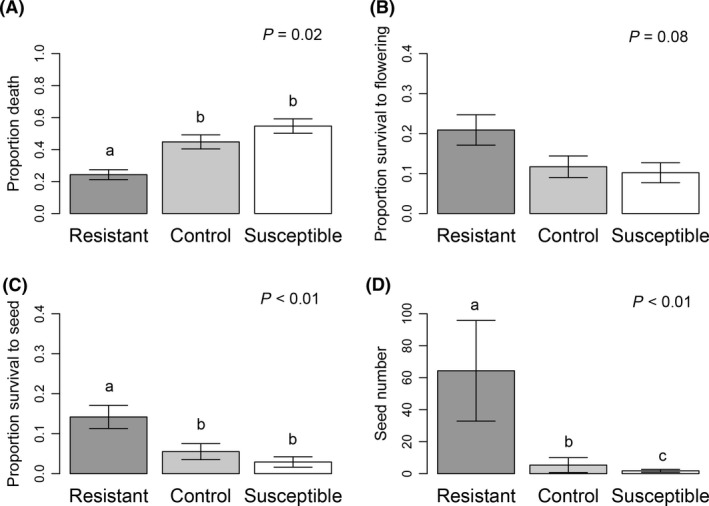
The proportion of individuals (±1 SE) following three generations of selection within each direction of selection (R/C/S) in the field that (A) died 2 weeks postherbicide application, (B) survived to set flowers and (C) seed. (D) The average number of seeds produced in the herbicide‐present environment. Significance of the adjusted pairwise comparisons between R/C/S is indicated above the bar.

Overall, 40.9% of individuals sprayed with herbicide in the field were dead 2 weeks postapplication of glyphosate (Fig. 1). Death differed significantly according to direction of selection (Selection effect, *χ* ^2^ = 7.817, *P* = 0.02; Table 1), with only 45.2% of susceptible individuals surviving 2 weeks postherbicide compared to 55.2% of control individuals and 75.6% of resistant individuals (Fig. 1) – these differences were significant when comparing survival between resistant and susceptible selection lines (G_1_ = 28.147; *P* < 0.001) and resistant and control lines (G_1_ = 13.773; *P* < 0.001), but not susceptible and controls (G_1_ = 2.776; *P* = 0.288). Of the experimental plants that survived, 14.4% set flower and 7.6% set seed. Survival to set flower was only marginally significantly different according to direction of selection (Fig. 1; selection effect, *χ* ^2^ = 4.983, *P* = 0.083; Table 1), however, the ability to set seed varied according to the direction of selection (Selection effect, *χ* ^2^ = 9.326, *P* < 0.001; Table 1). More individuals from the resistant lines produced seed in the herbicide‐present environment compared to nonselected control lines (Fig. 1C, 14.2% R vs. 5.5% C, respectively; G_1_ = 6.389; *P* = 0.03) and susceptible individuals (Fig. 1C, 5.3% S; G_1_ = 12.388; *P* = 0.001). The total number of seed produced by individuals sprayed with herbicide was low, but significantly differed according to direction of selection (Selection effect, *χ* ^2^ = 9.789, *P* = 0.007; Table 1), with individuals from R lines producing, on average, more seeds than S and C lines (Fig. 1D). These results show that the genetic lines selected for resistance in the greenhouse maintain these differences in survival in the field – and furthermore, lines selected for increased resistance exhibit a benefit of resistance by producing seed after being sprayed with glyphosate. We did not find variation among the replicate selection lines within each selection direction for survival or survival to produce seed, indicating that the response to selection for increased and decreased resistance was not due to genetic drift (Table 1). Although we identified a benefit of resistance, there was no evidence that resistance would respond to continued artificial selection in this study population – after three generations of selection, we found no family‐line variation for survival postspray or variation for the ability to produce seed in the presence of herbicide in the field (Table 1).

**Table 1:** The results from mixed models examining factors that underlie survival and the ability to set flower and seed, as well the number of seeds produced, in the field postglyphosate application (*N* = 430 plants). Values correspond to *χ* ^2^ from log‐likelihood tests with df = 1

	Survival		Set flower		Set seed		Number of seeds	
	*χ* ^2^	*P*	*χ* ^2^	*P*	*χ* ^2^	*P*	*χ* ^2^	*P*
Fixed effects								
Block	4.013	0.045	7.532	0.006	2.682	0.102	**8.871**	**0.003**
Selection	**7.817**	**0.020**	4.983	0.083	**9.326**	**0.009**	**9.789**	**0.007**
Random effects								
Family (Line)	0.000	1.000	0.157	0.692	0.050	0.824	0.283	0.595
Line (Selection)	1.669	0.196	0.000	1.000	0.000	1.000	0.000	1.000

Significant values are presented in bold.

### Cost of herbicide resistance

While individuals from R lines produced ~10% fewer flowers and 6% fewer seeds than susceptible individuals in the absence of herbicide, these differences were not significant, indicating that unlike the finding of a benefit of herbicide resistance, there is little cost of glyphosate resistance by this measure of fitness (Tables 2, 3). We did, however, find a significant effect of family for both traits (Table 3), indicating the presence of genetic variation for these traits within this experimental population. Selection for divergence in resistance likewise did not alter either the day of flowering of experimental plants or the average seed weight of progeny produced in the absence of herbicide (Table 2) – there was, however, a significant effect of selection replicate for both traits (Table 3).

**Table 2:** The results from mixed‐model analyses of variance examining factors that influence estimates of fitness and fitness correlates in the nonglyphosate environment (*N* = 425 plants). Values correspond to *χ* ^2^ from log‐likelihood tests with df = 1

	Day of flowering		Number flowers		Number seeds		Avg. seed weight	
	*χ* ^2^	*P*	*χ* ^2^	*P*	*χ* ^2^	*P*	*χ* ^2^	*P*
Fixed effects								
Block	3.509	0.061	**4.871**	**0.027**	**8.884**	**0.003**	0.031	0.862
Selection	2.075	0.354	2.281	0.320	1.211	0.546	3.411	0.182
Random effects								
Family (Line)	2.377	0.123	**5.119**	**0.024**	**4.521**	**0.033**	0.000	1.000
Line (Selection)	**4.386**	**0.036**	0.875	0.350	0.000	1.000	**7.343**	**0.007**

Significant values are presented in bold.

**Table 3:** Means (±1 SE) of fitness traits of plants grown in the absence of herbicide, summarized by direction of selection. Where appropriate, means that were significantly different after corrections are indicated by bolded lower‐case letters. Prop. Ng. Seeds = proportion nongerminated seeds

Trait	Resistant	Control	Susceptible
Day of flowering	63.42 ± 0.51	62.28 ± 0.52	61.73 ± 0.47
Early flower number	97.31 ± 4.86	95.1 ± 5.06	108.66 ± 5.51
Seed number	3144.75 ± 148.8	3100.02 ± 160.2	3332.67 ± 150.32
Seed weight	0.02 ± 0.001	0.02 ± 0.001	0.02 ± 0.001
Prop. germ. seeds	0.62 ± 0.04	0.55 ± 0.04	0.71 ± 0.03
Prop. ng. seeds	0.15 ± 0.02	0.13 ± 0.02	0.18 ± 0.02
Prop. dead seeds	0.23^**a**^ ± 0.03	0.31^**b**^ ± 0.04	0.11^**c**^ ± 0.02

Despite finding little evidence of a cost of resistance in the form of lower seed production, we found significant and notable differences in the proportion of dead seeds produced by individuals from divergent selection lines (Selection effect, *χ* ^2^ = 13.116, *P* < 0.001; Table 4), and marginal evidence that the proportion of germinable seeds differed across lines (Selection effect, *χ* ^2^ = 5.592, *P* = 0.06; Table 4). Individuals from resistant lines produced over twice as many nonviable seeds as susceptible individuals (23.4% R vs. 10.9% S; G_1_ = 60.344; *P* < 0.001, Table 3); control individuals likewise produced more nonviable seeds than susceptible individuals (31.4% C vs. 10.9% S; G_1_ = 132.811; *P* < 0.001, Table 3) and more nonviable seeds than resistant individuals (31.4% C vs. 23.4% R; G_1_ = 16.253; *P* < 0.001, Table 3). We found no evidence that the proportion of seeds that did not germinate and were not obviously inviable (i.e., are likely dormant) varied according to selection direction or maternal family (Table 3). There was no variation among replicate experimental trials for the seed traits (Table 3), nor was there evidence of variation between selection replicate within direction of selection. The latter finding indicates that the higher production of viable seeds produced by susceptible individuals was due to selection for decreased resistance and not due to genetic drift.

**Table 4:** The results from generalized linear mixed‐model analyses of variance examining factors that influence the proportion of dead and germinable seeds produced by maternal lines in the nonglyphosate environment (*N* = 240 plants). Values correspond to *χ* ^2^ from log‐likelihood tests with df = 1

Control only	Prop. dead seeds		Prop. germ. seeds		Neither germ. nor dead	
	*χ* ^2^	*P*	*χ* ^2^	*P*	*χ* ^2^	*P*
Fixed effects						
Replicate Experiment	1.342	0.247	0.623	0.430	0.007	0.936
Selection	**13.116**	**0.001**	5.592	0.061^	0.015	0.993
Random effects						
Family (Line)	1.371	0.242	**12.470**	**<0.001**	0.000	1.000
Line (Selection)	0.000	1.000	0.000	1.000	0.000	1.000

Significant values are presented in bold whereas an ^ indicates marginal significance.

## Discussion

Here, we present key findings for understanding glyphosate resistance evolution in the agricultural weed *I. purpurea*, the common morning glory. We first show that progeny from increased resistance lines exhibit a fitness benefit in the presence of herbicide in field conditions compared to control and susceptible lines. We likewise found evidence that susceptible individuals produce a greater number of germinable seeds than control and resistant individuals in the absence of herbicide, suggesting there is a potential cost associated with resistance alleles. Below, we discuss these points and place them in the context of other work investigating the evolutionary potential of glyphosate resistance in this and other systems.

### Benefit of herbicide resistance

*Ipomoea purpurea* has been commonly considered to exhibit low‐level resistance by weed managers for quite some time (considered “tolerance” in Culpepper 2006; see Baucom 2009 for discussion of terminology), but components necessary for understanding the evolutionary potential of this resistance have remained unexamined. Previously, we identified an additive genetic basis underlying resistance in this study population (Baucom and Mauricio 2008), and here, we further confirm through greenhouse crosses that resistance has a genetic basis. Our examination of progeny from the 3rd generation of divergent selection found that individuals selected for decreased resistance exhibited 45% survival postherbicide application in the field compared to 76% survival of the individuals from increased resistance lines. The differences in artificially selected lines were also evident in other fitness components, with more individuals from the R lines surviving to produce seeds in the field compared to the S and C lines postherbicide application. However, only 14% of individuals from the R lines produced seed, and seed production was overall very low in the herbicide‐present environment of this experiment. Thus, these while these lines exhibit differential resistance in the field at the rate of glyphosate applied, and our data again confirm that resistance in our study population is under genetic control (i.e., not due completely to the environment), we found that glyphosate application significantly reduced the seed production of the experimental *I. purpurea* plants in the field. Many studies that examine the level of herbicide resistance perform dose‐response experiments to report the percent of dry weight maintained following herbicide application (Powles et al. 1998; VanGessel 2001; Culpepper et al. 2006) and/or a visual estimate of the proportion “control” (VanGessel 2001; Culpepper et al. 2006). The current study was not designed in this manner (see Kuester et al. 2015 for a dose–response experiment) and as such we do not have dry biomass estimates that would allow us to compare the level of resistance between studies. However, we note that the survival of the R lines in our experiment (~76%) was lower than, but still comparable to, the reported estimate of survival of glyphosate resistant *Lolium rigidum* (~84% survival) sprayed with the same rate used in the study presented here (Powles et al. 1998).

Interestingly, after three generations of selection in the greenhouse, the difference in survival of resistance lines created from this single population is mirrored in the level of resistance among 44 populations of this species sampled from the landscape (Kuester et al. 2015). Our recent greenhouse assays show that, on average and at the rate of glyphosate used in the study presented here, 10 of 44 populations exhibit >70% survival, and another 10 exhibited <50% survival at this rate of herbicide (Kuester et al. 2015). Thus, after only three generations of artificial selection, the variation between R and S selection lines, sampled from the same base population in Georgia, was nearly as great as the variation among many natural populations sampled from Georgia to northern Ohio that have experienced selection via the herbicide for the past ~20 years (Kuester et al. 2015).

### Trade‐off between resistance and fitness

Fitness costs of resistance are equivocally documented across natural systems even though many studies report heritable variation in resistance. Interestingly, many studies investigating fitness costs of glyphosate resistance do not find evidence for them (Pedersen et al. 2007; Délye et al. 2013a; Vila‐Aiub et al. 2014) – this could be due to rapid compensatory evolution in the field, lack of control of the process of resistance evolution, or the examination of only a portion of a plant's life cycle (i.e., a focus on biomass vs. assessment of seed production and germination). Here, using a replicated artificial selection design, we find differences in some, but not all measured components of fitness in our study population. While the resistant lines produced 6% fewer seeds and ~10% fewer flowers than susceptible lines in the absence of herbicide in the field, these differences were not significant, and instead, evidence for the cost of resistance in this system appears to be associated primarily with seed germination and viability. If we weight the average number of seeds produced from individuals in the resistant and susceptible selection lines by the proportion of seeds that germinated in each, respectively, individuals from R lines produced ~19% fewer viable offspring than individuals from the S lines. We note that our germination assay used seeds that were open‐pollinated in the field, and thus, the differences that we identify are attributable to the selection history of the maternal background.

Although not commonly examined, germination differences associated with R and S biotypes have been identified in a handful of other weed species (reviewed in Vila‐Aiub et al. 2009). In the grass *Alopecurus myosuroides*, for example, fatal germination (seeds that germinate but show no further elongation of the root) was discovered in lines that segregate for mutations in the *ACCase* (acetyl‐coenzyme A carboxylase) gene, which endows resistance to ACCase herbicides (Délye et al. 2013b). As ACCase is an important enzyme in the production of fatty acids, the authors posited that mutations in *ACCase* could influence the natural lipid storage in the seed, which may influence both germination dynamics and seed viability (Délye et al. 2013b).

### What do fitness trade‐offs mean for herbicide resistance evolution?

The evolutionary influence of trade‐offs between defense and fitness is dependent, in part, on their genetic basis. If life‐history trade‐offs are due to linkage disequilibrium, the two traits exhibiting the trade‐off can quickly become evolutionarily independent, but if the trade‐off is due to pleiotropy, the genetic covariance between the traits may act as an evolutionary constraint (Futuyma 1998, 2010). If, in this system, the genetic variation underlying resistance and seed viability were independent (i.e., due to linkage disequilibrium), such variation would have to be tightly linked to cause an evolutionary constraint. One method used to discern linkage from pleiotropy as the source a life‐history trade‐off is by performing selection on traits that are correlated. Here, we selected on increases/decreases in resistance but not aspects of seed viability; thus, we cannot distinguish between linkage disequilibrium and pleiotropy as the source of the trade‐off. One of our results suggests linkage disequilibrium: while resistant maternal lines produced more dead seeds than susceptible lines when open pollinated in the field, the control lines produced significantly more dead seeds than resistant lines. This indicates that perhaps the allele producing the fitness cost is linked with the allele(s) conferring resistance, and more individuals in the control lines inherited this allele due to chance compared to the resistant and susceptible lines. The data do not rule out pleiotropy, however, and future work – either selecting in both directions on size and resistance, or genetic mapping of traits – will be required to better differentiate between pleiotropy and linkage as the basis of the identified trade‐off.

In conclusion, we used a balanced and replicated artificial selection design to show that this species exhibits fitness benefits and potential costs in the field according to the direction of selection. We found no evidence of detectable genetic variation within the selection lines following three generations of selection, which could suggest that the action of a single or very few genes confer resistance in this study population. Future work will examine the potential that the same fitness trade‐offs are replicated in natural, highly resistant populations collected across the landscape, and, as well, determine whether linkage or pleiotropy underlies the genetic basis of this cost.

## Conflict of Interest

There is no potential conflict of interest to declare.

## Data Archival Location

Data are available on Dryad and can be accessed via doi:10.5061/dryad.f098g.

## Supporting information


**Figure S1.** A diagram of the crossing design used in each generation of artificial selection.
