# Supplementary material for: An examination of fitness costs of glyphosate resistance in the common morning glory, Ipomoea purpurea
Source: Ecol Evol. 2015 Oct 26;5(22):5284–94. doi: 10.1002/ece3.1776 (PMC6102511; doi:10.1002/ece3.1776)
Supplement: Supplementary file 1 — Figure S1. A diagram of the crossing design used in each generation of artificial selection. [file ECE3-5-5284-s001.pdf]

Plant	1	2	3	4	5	6
7	X	X				
8	X	X	X			
9		X	X	X		
10			X	X	X	
11				X	X	X
12					X	X
